# Use of *International Classification of Diseases, Ninth Revision* Codes for Obesity: Trends in the United States from an Electronic Health Record-Derived Database

**DOI:** 10.1089/pop.2017.0092

**Published:** 2018-06-01

**Authors:** Michelle Mocarski, Ye Tian, B. Gabriel Smolarz, John McAna, Albert Crawford

**Affiliations:** ^1^ Novo Nordisk, Inc., Plainsboro, New Jersey.; ^2^ College of Population Health, Thomas Jefferson University, Philadelphia, Pennsylvania.

**Keywords:** obesity coding, electronic health records, prevalence

## Abstract

Obesity is a potentially modifiable risk factor for many diseases, and a better understanding of its impact on health care utilization, costs, and medical outcomes is needed. The ability to accurately evaluate obesity outcomes depends on a correct identification of the population with obesity. The primary objective of this study was to determine the prevalence and accuracy of *International Classification of Diseases, Ninth Revision* (ICD-9) coding for overweight and obesity within a US primary care electronic health record (EHR) database compared against actual body mass index (BMI) values from recorded clinical patient data; characteristics of patients with obesity who did or did not receive ICD-9 codes for overweight/obesity also were evaluated. The study sample included 5,512,285 patients in the database with any BMI value recorded between January 1, 2014, and June 30, 2014. Based on BMI, 74.6% of patients were categorized as being overweight or obese, but only 15.1% of patients had relevant ICD-9 codes. ICD-9 coding prevalence increased with increasing BMI category. Among patients with obesity (BMI ≥30 kg/m^2^), those coded for obesity were younger, more often female, and had a greater comorbidity burden than those not coded; hypertension, dyslipidemia, type 2 diabetes mellitus, and gastroesophageal reflux disease were the most common comorbidities. Key findings: US outpatients with overweight or obesity are not being reliably coded, making ICD-9 codes undependable sources for determining obesity prevalence and outcomes. BMI data available within EHR databases offer a more accurate and objective means of classifying overweight/obese status.

## Introduction

Obesity has become one of the most important public health challenges of the modern era. Globally, the prevalence of obesity has grown at an alarming rate over recent decades, and it has more than doubled since 1980.^1^ Data from the National Health and Nutrition Examination Survey, 2011–2012, showed that more than one third of adults in the United States – more than 78 million individuals – were obese, with the prevalence being higher in middle-aged than younger adults.^2^ The medical consequences of obesity include multiple comorbidities such as type 2 diabetes mellitus, fatty liver disease, hyperlipidemia, hypertension, coronary artery disease, obstructive sleep apnea, and osteoarthritis, among others,^3–6^ adding to the morbidity, mortality, and economic burden of obesity.^7–9^ Given that obesity is a potentially modifiable risk factor, there is an urgent need to develop a greater understanding of its relationships to other diseases and impact on health care utilization, cost, and medical outcomes. In other disease states, this often is analyzed in the context of claims data generated from *International Classification of Diseases* (ICD) coding of primary diseases, complications, and interventions. However, if overweight and obesity are not reliably coded in these databases, then the study of the subsequent complications and interventions will be limited and potentially biased.

In order to study the nature and scope of obesity-related complications, whether it be from a medical or payer perspective, it is critical that the population being studied is identified accurately and completely for the findings to be valid. Although medical claims data are well recognized as useful for large-scale evaluations of disease epidemiology, including medical and economic outcomes, previous research has demonstrated that coding for obesity and body mass index (BMI) in medical claims is inconsistent at best and is significantly underrepresentative of populations with overweight or obesity.^10–16^ The reasons are numerous, but it is likely that ICD-9 coding for overweight and obesity is often overlooked unless it is the primary reason patients are seeking treatment. In addition, there are provider reimbursement challenges for obesity management, thus making the reimbursement code alone not well suited to identifying patients who may be overweight or obese.

Increased adoption of electronic health record (EHR) technology by physicians in the United States is providing a new source of health care data for outcomes research purposes. Such data are based on information actually measured during the routine process of patient care and include biometric data, such as BMI, not available in claims databases. The Quintiles electronic medical record (EMR; QuintilesIMS, Durham, NC) research database is a commercially-available, high-quality source of anonymized patient-level ambulatory medical records. This database captures a range of demographic and clinical variables, including BMI, and is increasingly being used for outcomes research pertaining to various disease states.^17–22^

The primary objective of this study was to determine the prevalence and accuracy of ICD-9 coding for obesity and overweight within the Quintiles EMR, compared to BMI values calculated from patient data. The study also was designed to analyze characteristics of patients with overweight or obesity in the database (based on BMI values) who did or did not receive ICD-9 codes for overweight or obesity. A third objective was to examine whether the use of ICD-9 coding for overweight and obesity changed between 2011 and 2014.

## Methods

### Overall study design and data collection

This was a cross-sectional, 2-part analysis of data from a US primary care EMR database, Quintiles EMR. The Quintiles EMR database is used in the ambulatory care setting, with more than 1300 sites in 49 states. There are more than 30,000 clinicians using the system, resulting in approximately 35 million patients in the database. Source data for the Quintiles EMR database are captured with the GE Centricity (GE Healthcare, Chicago, IL) user interface. Participating physicians are in middle- to large-size group practices, with approximately 85% being primary care providers (eg, family practice, internal medicine, obstetrics/gynecology, pediatrics). Geographic areas served by the Quintiles EMR database are similar to the overall US population and demographic characteristics are similar to utilizers of health care in the National Ambulatory Medical Care Survey.^23^ This database may or may not document care provided in other health care sectors, such as inpatient services and procedures (eg, imaging, same-day surgery, acute rehabilitation, long-term care).

Demographic data collected from the Quintiles EMR database included patient age, sex, race, and geographic region of the United States. BMI measurements were determined from actual patient height and weight values in the database as input by clinical staff. Weight category classifications (underweight, normal, overweight, Class I-III Obesity) were based on BMI cutoffs set by the National Heart, Lung, and Blood Institute. These analyses were limited to adult patients aged 20 years and older.

ICD-9 codes are comprised of 3, 4, or 5 digits, with the first 3 digits being mandatory. Greater specificity is achieved by the addition of 1 or 2 additional digits following a decimal place. The following ICD-9 codes for overweight/obesity were used in this study: 278 (Overweight, obesity and other hyperalimentation); 278.0 (Overweight and obesity); 278.00 (Obesity unspecified); 278.01 (Morbid obesity); ICD-9 278.02 (Overweight); and 278.03 (Obesity hypoventilation syndrome). Patients with claims evidence of pregnancy or gestational diabetes were not included because of expected BMI fluctuations related to pregnancy.

#### Part I: Prevalence and characteristics of patients receiving ICD-9 codes for overweight/obesity

The primary analysis was a point-in-time assessment of the prevalence of ICD-9 coding for patients with overweight and obesity in the Quintiles EMR database and comparative characteristics of coded versus non-coded groups. The inclusion criteria for patients included in this analysis were: available BMI measurement in the Quintiles EMR database between January 1, 2014, and June 30, 2014; ≥20 years of age on index date; no evidence of pregnancy or gestational diabetes; and at least 3 months of follow-up data after the index BMI (first recorded BMI during the study period) to provide an adequate time window for capturing ICD-9 coding.

All 3-month pre-index and 3-month post-index medical records were searched for ICD-9 codes for overweight and obesity. The rationale for searching 3 months before and after the index BMI was to search for a sufficiently long interval for the provider to have responded to the patient's increased weight, but not so long that the patient's weight was likely to have changed substantially. The proportions of patients coded for overweight and obesity by ICD-9 codes were compared with the proportions of patients in corresponding weight categories based on actual height and weight measurements. Among patients with BMI-categorized obesity (BMI ≥30 kg/m^2^), characteristics of age, sex, race, region of the United States, and comorbidities were compared between subgroups with and without ICD-9 codes for overweight/obesity. Comorbidities were determined based on ICD-9 codes. The Charlson comorbidity index (CCI), a method widely used to measure the burden of comorbid disease and predict mortality,^24^ was calculated. This index consists of a list of 17 diagnoses and assigns a weight from 1 to 6 to each diagnosis. The summary score is the sum of weighted values.

Descriptive statistics for all study variables were calculated. Categorical variables are described using the proportion of patients in each category, and continuous variables are described using mean and standard deviation. Logistic regression was conducted to obtain odds ratios (ORs) for receiving an ICD-9 code for overweight or obesity.

#### Part II: Coding trends over time

This analysis compared 4 sequential years of coding patterns using data from January 1, 2011, to September 30, 2014. Patients for whom index BMI values were available during each calendar year (2011–2014) were grouped into conventional BMI categories, and the database searched for any ICD-9 codes for obesity or overweight over the whole year. The annual prevalence of coding was examined by BMI category and compared across the years. This analysis was performed on all patients with BMI data, and for male and female subsets separately.

## Results

### Part I: Prevalence and characteristics of patients receiving ICD-9 codes for overweight/obesity

The study sample for this analysis included 5,512,285 patients with any BMI value recorded between January 1, 2014, and June 30, 2014. According to BMI values, 74.6% of the study cohort had a BMI ≥25.0 and thus were eligible to be coded for overweight or obesity. However, only 15.1% of all patients (n = 833,763) had ICD-9 codes for overweight or obesity.

The most commonly used ICD-9 code was 278.00 (Obesity, unspecified) in 10.6% of patients, followed by 278.02 (Overweight) in 2.6% of patients, and 278.01 (Morbid obesity) in 2.5% of patients. Codes 278.0 (Overweight and obesity), 278 (Overweight, obesity, and other hyperalimentation), and 278.03 (Obesity hypoventilation syndrome) were each used in <1% of patients.

#### Overweight and obesity coding by BMI category

Of all patients with an “overweight” BMI (25.0–29.9 kg/m^2^; 32.3% of the population), 3.86% were correctly coded for overweight only, 2.79% were incorrectly coded for obesity only, and 0.19% coded for both obesity and overweight (Table 1). Of all patients with an “obese” BMI (≥30.0 kg/m2; 42.3% of the population), 27.3% were correctly coded for obesity only, 2.3% were incorrectly coded for overweight only, and 0.4% were coded for both obesity and overweight.

**Table 1.** Prevalence of Overweight/Obesity-Related Coding for Patients ≥20 Years Old Within Body Mass Index Categories (for Patients Who Have Index Body Mass Index Measurements)

			*ICD-9 coding for obesity/overweight,* n *(% of patients in BMI category/row)*		
*BMI category*	*BMI (kg/m^2^)*	*Patients with index BMI,* n *(total* N* = 5,512,285)*	*Diagnosis of obesity only*^a^	*Diagnosis of overweight only*^b^	*Diagnosis of both obesity*^a^*and overweight*^b^
Underweight	<18.5	72,683	352 (0.48)	76 (0.10)	10 (0.01)
Normal	18.5–24.9	1,324,866	4954 (0.37)	5434 (0.41)	199 (0.02)
Overweight	25.0–29.9	1,782,522	49,780 (2.79)	68,878 (3.86)	3475 (0.19)
Obese Class I	30.0–34.9	1,233,966	207,037 (16.78)	35,540 (2.88)	5295 (0.43)
Obese Class II	35.0–39.9	618,586	192,837 (31.17)	12,814 (2.07)	1933 (0.31)
Obese Class III	40.0–44.9	279,186	122,066 (43.72)	4239 (1.52)	943 (0.34)
	45.0–49.9	117,668	62,435 (53.06)	1425 (1.21)	384 (0.33)
	≥50.0	82,808	52,822 (63.79)	582 (0.70)	253 (0.31)

*Shaded cells* designate correct coding according to recorded BMI.

^a^ ICD-9 278 (Overweight, obesity and other hyperalimentation); ICD-9 278.0 (Overweight and obesity); ICD-9 278.00 (Obesity unspecified), ICD-9 278.01 (Morbid obesity), ICD-9 278.03 (Obesity hypoventilation syndrome).

^b^ ICD-9 278.02 (Overweight).

BMI, body mass index; ICD-9, *International Classification of Diseases, Ninth Revision*.

The percentage of patients coded with an accurate ICD-9 code increased with increasing BMI category, with approximately two thirds of subjects in the highest BMI category (≥50 kg/m^2^) having a correct ICD-9 code for obesity (278.00, Obesity, unspecified; 278.01, Morbid obesity; 278.03, Obesity hypoventilation syndrome) and less than 1% of patients in this BMI category having a code for overweight (278.02). In the lowest BMI category for obesity (30–34.9 kg/m^2^), 17% of patients were coded for obesity. The ICD-9 code for morbid obesity (278.01) appeared to be applied most accurately, with a majority (70.9%) of its use occurring in patients with BMI ≥40 kg/m^2^.

Table 2 presents the demographics of the subgroup of study patients with an index BMI ≥30 kg/m^2^, as well as comparative demographics for those coded for obesity and those who were not. The patients who were coded for obesity (28.0% of eligible patients), were younger, more often female, and had a greater comorbidity burden compared with patients not coded for obesity but who were obese as assessed by actual BMI. Among patients with BMI ≥30 kg/m^2^, the mean (±SD [standard deviation]) CCI score was higher among patients coded for obesity than for those not coded for obesity. Even among patients in the highest CCI score category (5+), only 37% were coded for obesity.

**Table 2.** Baseline Characteristics Among Patients with Index Body Mass Index ≥30 kg/m^2^ According to Obesity Coding Status

	*Patients with BMI ≥30 kg/m^2^*			
	*All*	*Diagnosis of obesity,* N *(% of row)*	*No diagnosis of obesity,* N *(% of row)*	P *value*^a^
Total, n	2,332,214	646,005 (28.0)	1,686,209 (72.0)	
Age				
Mean (SD)	54.9 (14.9)	53.2 (14.4)	55.6 (15.0)	<.0001
Age group, n (%)				
20–44	586,201	181,779 (31.0)	404,422 (69.0)	<.0001
45–64	1,060,785	307,604 (29.0)	753,181 (71.0)	
≥65	685,228	156,622 (22.9)	528,606 (77.1)	
Sex, n (%)				
Female	1,360,468	406,958 (29.9)	953,510 (70.1)	<.0001
Male	971,652	239,022 (24.6)	732,630 (75.4)	
Unknown	94	25 (26.6)	69 (73.4)	
Race, n (%)				
White	1,632,875	428,588 (26.2)	1,204,287 (73.8)	<.0001
Black	260,012	89,401 (34.4)	170,611 (65.6)	
Hispanic	44,037	16,680 (37.9)	27,357 (62.1)	
Asian	18,608	4979 (26.8)	13,629 (73.2)	
Native American	2378	1032 (43.4)	1346 (56.6)	
Multi	10,107	4275 (42.3)	5832 (57.7)	
Other	19,841	5484 (27.6)	14,357 (72.4)	
Unknown/Undetermined	329,566	92,315 (28.0)	237,251 (72.0)	
Not entered	14,790	3251 (22.0)	11,539 (78.0)	
Region, n (%)				<.0001
Midwest	464,053	125,369 (27.0)	338,684 (73.0)	
Northeast	603,127	196,231 (32.5)	406,896 (67.5)	
South	886,581	224,944 (25.4)	661,637 (74.6)	
West	378,453	99,461 (26.3)	278,992 (73.7)	
BMI, kg/m^2^				
Mean (SD)	36.3 (6)	39.2 (7.2)	35.2 (5.0)	<.0001
CCI Score				
Mean (SD)	0.8 (1.3)	0.9 (1.4)	0.7 (1.2)	<.0001
CCI Category, n (%)				
0	1,371,705	334,202 (24.4)	1,037,503 (75.6)	<.0001
1	526,363	169,846 (32.3)	356,517 (67.7)	
2	216,336	65,589 (30.3)	150,747 (69.7)	
3	109,954	37,108 (33.8)	72,846 (66.3)	
4	50,735	18,378 (36.2)	32,357 (63.8)	
5+	57,121	20,882 (36.6)	36,239 (63.4)	

^a^ *P* values for coded vs non-coded patients; *t* test for continuous variables, chi-square test for categorical values.

BMI, body mass index; CCI, Charlson comorbidity index; SD, standard deviation.

The mean BMI was significantly higher in the coded versus the non-coded group, but it was still notably high in the non-coded group (Table 2). Geographically, the highest proportion of coded patients was observed in the Northeast region of the United States. By race category, the highest proportion of coding occurred among patients classified as Native American and Multiethnic. White patients comprised the greatest percentage of patients in the population with BMI ≥30 kg/m^2^, yet the prevalence of obesity coding among them was the lowest of the major racial groups evaluated.

Baseline characteristics for patients with an index BMI of 25–29.9 kg/m^2^ and whether or not they were coded for overweight are provided in online Supplementary Table S1 (Supplementary Data are available online at www.liebertpub.com/pop).

#### Coding and specific comorbidities

The most common comorbidities among patients with a BMI ≥30 kg/m^2^ were hypertension, dyslipidemia, type 2 diabetes mellitus, and gastroesophageal reflux disease (Table 3). Among patients coded for these comorbidities, the prevalence of obesity coding concomitant with these diagnoses ranged from 32.5% to 36.9%.

**Table 3.** Prevalence of Select Comorbidities and Obesity Coding Prevalence, Patients with Body Mass Index ≥30 kg/m^2^ and Existing Comorbidities at Index Body Mass Index^a^

		*Patients with BMI ≥30 kg/m^2^ (*n* = 2,332,214)*		
*Comorbidity*	n	*Comorbidity*^b^*prevalence, %*	*Prevalence of obesity coding*^c^*within comorbidity category, %*	P *value*^d^
Any CVD	1,161,465	49.8	31.3	<.0001
—Hypertension	991,773	42.5	32.5	<.0001
—Other CVD	588,676	25.2	29.6	<.0001
Dyslipidemia	933,876	40.0	32.8	<.0001
T2DM	454,757	19.5	36.9	<.0001
GERD	369,652	15.8	33.4	<.0001
Depression	307,436	13.2	36.9	<.0001
Malignancy	285,811	12.3	28.9	<.0001
Osteoarthritis	278,303	11.9	31.0	<.0001
Vitamin D Deficiency	225,072	9.7	39.6	<.0001
Sleep Apnea	212,157	9.1	46.0	<.0001
Prediabetes	124,287	5.3	40.4	<.0001
Chronic Kidney Disease	108,881	4.7	34.3	<.0001
NAFLD	34,587	1.5	44.9	<.0001
Gallbladder Disease	32,212	1.4	35.9	<.0001
Metabolic Syndrome	18,325	0.8	52.2	<.0001
Dyspepsia	16,971	0.7	33.6	<.0001
Inflammatory Bowel Disease	13,352	0.6	23.5	<.0001
HIV	6242	0.3	31.8	<.0001
Acute/Chronic Pancreatitis	5765	0.2	29.1	0.0143
Anorexia	1788	0.1	25.0	0.0108
Cushing Syndrome	828	0.04	44.3	<.0001
Feeding Difficulties	117	0.005	23.1	0.2653
Prader-Willi Syndrome	92	0.004	55.4	<.0001
Cachexia	50	0.002	18.0	0.1303

^a^ Index BMI = first recorded BMI measurement during the study period.

^b^ Comorbidity confirmed by existing diagnosis ±3 month window around index BMI.

^c^ ICD-9 codes 278, 278.0, 278.00, 278.01, 278.03.

^d^ *P* values for coded vs non-coded patients; *t* test for continuous variables, chi-square test for categorical variables.

BMI, body mass index; CVD, cardiovascular disease; GERD, gastroesophageal reflux disease; HIV, human immunodeficiency virus; ICD-9, *International Classification of Diseases, Ninth Revision*; NAFLD, nonalcoholic fatty liver disease; T2DM, type 2 diabetes mellitus.

In contrast, comorbidities such as Prader-Willi Syndrome, metabolic syndrome, sleep apnea, nonalcoholic fatty liver disease, and Cushing syndrome were coded much less frequently (Table 3). However, patients with these comorbid conditions were more likely to also be accurately coded for obesity (44.3% to 55.4%).

Corresponding data for patients with an index BMI of 25–29.9 kg/m^2^ (overweight) are provided in online Supplementary Table S2.

Table 4 provides logistic regression model estimates for probabilities of being coded for obesity according to demographic and clinical characteristics. Younger adults had a higher probability of being coded compared with older adults, and female sex was associated with an OR of about 1.3 relative to male sex. Native American, Multiracial, Hispanic, and black patients had higher ORs for being coded relative to white patients. The probability of being coded for obesity increased with increasing CCI category. Specific comorbidities with the highest ORs included Prader Willi Syndrome, metabolic syndrome, and sleep apnea.

**Table 4.** Odds Ratios and 95% Confidence Intervals from Logistic Regression Model Estimating the Probability of Being Coded for Obesity, Patients with Body Mass Index ≥30 kg/m^2^

	*Odds ratio*	*95% CI*	P *value*^a^
Demographic Characteristics			
Age (ref = 65+)			
20–44	1.937	1.917–1.956	<.0001
45–64	1.458	1.446–1.470	<.0001
Female (ref = Male)	1.339	1.329–1.348	<.0001
Race (ref = White)			
Asian	0.988	0.955–1.021	<.0001
Black	1.438	1.424–1.452	<.0001
Hispanic	1.691	1.657–1.726	<.0001
Native American	2.173	1.999–2.362	<.0001
Multi	1.799	1.727–1.873	<.0001
Other	1.053	1.020–1.088	<.0001
Geographic Location (ref = West)			
Midwest	1.048	1.036–1.060	0.0192
Northeast	1.253	1.240–1.267	<.0001
South	0.946	0.936–0.955	<.0001
Clinical characteristics			
CCI Category (ref = 0)			
1	1.226	1.215–1.236	<.0001
2	1.239	1.224–1.255	<.0001
3	1.420	1.396–1.444	<.0001
4	1.593	1.556–1.631	<.0001
5+	1.713	1.671–1.757	<.0001
Comorbidities			
Prader Willi Syndrome	2.245	1.417–3.556	0.0006
Metabolic Syndrome	2.194	2.124–2.267	<.0001
Sleep Apnea	2.164	2.141–2.186	<.0001
Prediabetes	1.523	1.503–1.544	<.0001
NAFLD	1.515	1.478–1.552	<.0001
Cushing Syndrome	1.366	1.170–1.596	<.0001
Vitamin D Deficiency	1.333	1.319–1.347	<.0001
T2DM	1.241	1.228–1.253	<.0001
Hypertension	1.237	1.228–1.247	<.0001
Dyslipidemia	1.211	1.202–1.221	<.0001
Depression	1.234	1.222–1.245	<.0001
Gallbladder Disease	1.170	1.140–1.201	<.0001
Osteoarthritis	1.078	1.068–1.089	<.0001
Feeding Difficulties	1.065	0.669–1.693	0.7913
Dyspepsia	1.036	0.998–1.075	0.0632
CVD	0.928	0.921–0.936	<.0001
Chronic Kidney Disease	0.910	0.894–0.926	<.0001
Malignancy	0.866	0.858–0.875	<.0001
Acute/Chronic Pancreatitis	0.806	0.757–0.858	<.0001
Inflammatory Bowel Disease	0.741	0.709–0.775	<.0001
Anorexia	0.740	0.658–0.832	<.0001
HIV	0.665	0.621–0.712	<.0001
Cachexia	0.440	0.191–1.015	0.0541

^a^ From regression analysis.

CCI, Charlson comorbidity index; CI, confidence interval; CVD, cardiovascular disease; HIV, human immunodeficiency virus; NAFLD, nonalcoholic fatty liver disease; T2DM, type 2 diabetes mellitus.

Similar patterns were observed for coding of overweight (online Supplementary Table S3), although CCI category had much less influence on the probability of being coded. The comorbidities with the highest ORs for overweight coding were HIV, metabolic syndrome, and prediabetes.

### Part II: Coding trends over time

**FIG. 1.**
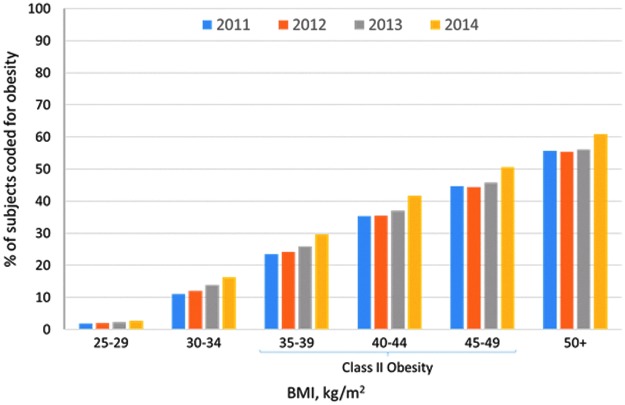
Prevalence of coding for obesity^a^ by index body mass index^b^ category, by year, 2011 to 2014.^c^ ^a^ICD-9 codes (278, 278.0, 278.00, 278.01, 278.03) captured ±3 months from date of index BMI in each year. ^b^Index BMI = first recorded BMI measurement during the study period. ^c^2014 data reflect 9 months (January – September). BMI, body mass index; ICD-9, *International Classification of Diseases, Ninth Revision.*

Among patients with BMI ≥30 kg/m^2^, the prevalence of ICD-9 coding for obesity increased slightly each year from 2011 to 2014 (Fig. 1), with a slightly greater increase apparent between 2013 and 2014 than between other years, particularly within BMI categories ≥35 kg/m^2^. For all years, the prevalence of coding for obesity was low, but it increased as BMI category increased. Similar trends were observed for both males and females, though the prevalence of coding was consistently higher among women than men (Table 5). Similar trends were observed for coding among males and females even when analyzed by age categories (data not shown).

**Table 5.** Prevalence of *International Classficiation of Diseases, Ninth Revision* Coding for Obesity^a^ by Index Body Mass Index^b^ by Year and Sex

	*% of patients with ICD-9 codes for obesity*^a^							
	*2011*		*2012*		*2013*		*2014*	
*BMI, kg/m*^2^	*Females*	*Males*	*Females*	*Males*	*Females*	*Males*	*Females*	*Males*
25.0–29.9	2.4	1.3	2.5	1.5	2.7	1.6	3.3	2.0
30.0–34.9	12.1	9.8	13.2	10.7	15.2	12.4	17.9	14.4
35.0–39.9	23.7	23.3	24.5	23.8	26.4	25.2	30.4	28.8
40.0–44.9	34.7	36.7	35.0	36.6	36.5	37.9	41.4	42.2
45.0–49.9	43.4	47.9	43.3	46.6	44.7	48.2	49.8	52.5
≥50.0	54.5	58.9	54.2	58.1	55.1	58.7	59.9	63.4

^a^ ICD-9 codes 278, 278.0, 278.00, 278.01, 278.03.

^b^ Index BMI = first recorded BMI measurement during the study period.

BMI, body mass index; ICD-9, *International Classficiation of Diseases, Ninth Revision.*

Similar trends were observed for coding for overweight, though coding for overweight was observed in very low proportions of patients overall (online Supplementary Fig. S1). Among patients with a BMI consistent with the classification of overweight (BMI 25–29 kg/m^2^), the proportion of patients with ICD-9 coding for overweight was very low: 1.8% in 2011, 2.4% in 2012, 3.4% in 2013, and 3.9% in 2014. Prevalence of ICD-9 coding for overweight by year and sex is provided in online Supplementary Table S4.

## Discussion

This study provided a unique opportunity to examine the prevalence and trends associated with clinician ICD-9 coding for overweight and obesity based on actual patient BMI data. Results demonstrate that ICD-9 codes should not be relied on to estimate population prevalence rates of obesity or overweight, given the likelihood of capturing only a portion of relevant patients and generating inaccurate conclusions about the magnitude of the obesity problem. Furthermore, even in other conditions where weight may be an effect modifier, using ICD-9 codes for obesity/overweight will not provide an effective control of confounding.

Even for patients in the very highest BMI categories of 40 kg/m^2^ and greater only one third to just over one half were coded for obesity in any given year. Although the frequency of coding for all categories of overweight or obesity increased each year of the study, the overall frequency remained extremely low for such an important disease.

Interestingly, patients with obesity with rarely coded comorbidities such as Prader-Willi Syndrome, metabolic syndrome, sleep apnea, nonalcoholic fatty liver disease, and Cushing syndrome were the most likely to receive codes for obesity. Among this group, Prader-Willi Syndrome and Cushing Syndrome are epidemiologically uncommon, occurring very rarely in any population and are specialist treated. The fact that obesity is a core presentation of both of these conditions may increase its likelihood for coding. In contrast, the other comorbid conditions where obesity coding is common, such as metabolic syndrome, sleep apnea, and nonalcoholic fatty liver disease are actually epidemiologically quite common; however, they are also commonly underdiagnosed and, thus, likely to also be undercoded. In the case where one of these comorbidities has been diagnosed and is being treated, coding alongside obesity coding may not be surprising, as each of these comorbidities is driven by obesity. Additionally, because it is not common to code for these comorbidities or for obesity, it is possible that there are particular clinicians who are more likely to either assess these conditions and/or code for them. Surprisingly, the rate of obesity coding was lower among other obesity-driven comorbidities such as type 2 diabetes, hypertension, and dyslipidemia. However, perhaps their high population prevalence, in addition to the comorbid presentation of all 3, does not provide an automatic trigger to clinicians to also code for obesity.

Possible reasons for incomplete obesity and overweight coding might include the time burden of documentation, including prioritizing other codes in the limited time visit, shortage of time to search for the code, lack of knowledge of the existence of ICD codes for overweight and obesity, EHR system issues wherein the codes do not come up in searches, the limit of only 4 codes per procedure/visit (higher prioritization for “mainstream” diseases), and a nihilistic view of coding weight status related to a perception or reality of limited or no reimbursement for management of obesity, because there currently is no increased payment or minimal economic incentive to do so with many payers. It is also worth noting that the data found an unexpected “false positive” phenomenon in which a small percentage of patients with very low BMIs (<25 kg/m^2^) were coded as overweight or obese, which could suggest a certain level of operational or administrative error when it comes to coding accuracy in general.

A lack of obesity coding behavior also may reflect a general aversion among clinicians toward addressing the topic of weight with their patients. Reasons for such behavior can include time limitations, failure to prioritize obesity as a clinical issue, negative stereotypes and attitudes about people with obesity, low levels of emotional rapport with people with obesity, and poor expectations for weight loss success.^25–28^ Some of these challenges may be addressed by improving health care provider training in weight management.

Another likely phenomenon of the obesity epidemic is that, as the mean BMI in the US has risen, patients with BMIs >25 kg/m^2^ (overweight) or >30 kg/m^2^ (obese) are subconsciously assessed as reflecting the norm rather than a population at increased health risk that requires intervention. In addition, clinicians are likely unaware of the biological basis of obesity, and hence may be cynical about their ability to facilitate weight loss. This is probably driven by past disappointments with diet and exercise interventions or historic failures of older weight loss medications. Data from the National Ambulatory Medical Care Survey revealed a significant decline in primary care physician-based weight counseling efforts from 1995–1996 to 2007–2008 in the United States.^29^ This is an alarming trend, given the millions of individuals with overweight/obesity, which has well-established health consequences such as diabetes, heart disease, fatty liver, and others, and the fact that overweight/obesity is a modifiable risk factor.

A number of major changes have occurred in recent years with potential impact on the medical coding environment in the United States. The US Health Information Technology for Economic and Clinical Health Act, passed in 2009, provides financial incentives to hospitals and outpatient clinics that utilize EHRs meeting certain requirements, known as “meaningful use” standards. As part of these standards, BMI calculations must be included in the EHRs. Adoption of EHR compliance among outpatient providers reportedly rose from 17% in 2006 to 78% in 2013.^30^ The Affordable Care Act (ACA) went into full effect in January, 2014, providing millions of new patients access to the US health care system overall, though adding additional administrative burdens to medical practices as they adjust to greater patient demand, multitudes of regulations, and adapt to additional payer models. However, the ACA also has strict documentation rules, reinforcing the need for medical coding accuracy to avoid denial of reimbursement. The current data noted a slight but noticeable increase in obesity coding prevalence in 2014 relative to other years, most evident among patients with BMI ≥35. Although this finding coincides with the full implementation of the ACA in the same calendar year, possible causal associations cannot be determined. The ACA does require full coverage for preventive care obesity screening and counseling by most insurers, possibly increasing the financial incentive for physicians to code and bill for weight-related services. Further studies looking at coding trends over time will be needed to evaluate the ongoing impact of the ACA and Medicare and commercial payer quality reporting requirements on coding compliance and accuracy.

Another change affecting the US health care system, and which the current data do not reflect, is the implementation of the ICD-10 coding system in October 2015. The ICD-10 system represents a tremendous expansion of coding complexity, as it contains approximately 72,000 diagnosis codes compared with 14,000 in the ICD-9 system. Previous numeric ICD codes for diseases have been replaced with a vastly expanded set of alphanumeric codes. Although the intention is greater specificity and accuracy, it remains to be seen if this new system will achieve the desired goals given the much greater effort on behalf of the clinical team to search, identify, and document these new codes. The present data may provide a useful benchmark for future studies to examine obesity coding trends with the ICD-10 system.

Previous US studies of varying methodology and sample size have reported under-recognition and poor coding practices for obesity, including studies utilizing Medicare records,^31^ the Mayo Clinic Rochester primary care database,^13^ and US military health system EMRs.^14^ The present data corroborate and expand on these prior findings, providing additional robust evidence from a multimillion patient sample with broad national representation of outpatient coding practices in the United States. The present findings add to a growing body of evidence substantiating the critical limitations of relying on medical coding for tracking obesity burden, epidemiology, expenditures, and outcomes.

Although not all providers will accept and enact the requirement, as of January 2014 all health care providers in the United States are now federally mandated to use EHR systems. Hopefully, this will increase the scope and utility of such databases for outcomes research, though it is unclear to what extent researchers will have access to these improved data sets.

What should be equally concerning about these data is the implication that if clinicians are not coding obesity, they likely are not treating obesity^13^; prior research has revealed that documentation of obesity correlates with interventional recommendations.^32^ Given that two thirds of the US population is overweight or obese, the medical community needs to proactively embrace treating overweight and obesity much as they do for other chronic diseases such as depression or hypertension. In fact, the magnitude of the problem represents a tremendous opportunity for clinicians to make an impact on numerous weight-related diseases simply by maintaining awareness of obesity as a disease and being proactive in recommending evidence-based and effective weight-loss measures. Proactive physician communication, intervention, and advice have been shown to be effective motivators for weight-loss behavior,^33^ and medical therapy is often indicated.

## Conclusions

This analysis found that patients with measured BMIs indicating overweight or obese status in outpatient settings in the United States still are not reliably coded in claims data as such. Physicians, payers, and administrators who are dedicated to evaluating medical and economic outcomes related to obesity must be aware that medical coding data for overweight and obesity are wholly inadequate for these purposes. BMI determinations from clinical height and weight data available within EHR databases are, in theory, easily accessible and provide an accurate, objective means of classifying overweight and obesity status. The apparent high degree of discordant obesity coding may signal a need for efforts to correct underlying perceptions and attitudes of physicians regarding the medical relevance of obesity and the need for effective intervention.

## Supplementary Material

Supplemental data

Supplemental data

Supplemental data

Supplemental data

Supplemental data
