# Supplementary material for: Use of International Classification of Diseases, Ninth Revision Codes for Obesity: Trends in the United States from an Electronic Health Record-Derived Database
Source: Popul Health Manag. 2018 Jun 1;21(3):222–30. doi: 10.1089/pop.2017.0092 (PMC5984561; doi:10.1089/pop.2017.0092)
Supplement: Supplemental data [file Supp_Table1.pdf]

Supplementary Table S1. Baseline Characteristics Among Patients with Index Body Mass Index 25–29 kg/m^2 (Overweight) According to Overweight Coding Status

	Patients with BMI 25–29 kg/m^2			
	All	*Diagnosis of overweight,* N *(% of row)*	*No diagnosis of overweight* N *(% of row)*	P *value*[a]
Total, n	1,782,522	72,353	1,710,169	
Age				
Mean (SD)	57.0 (16.4)	53.0 (15.4)	57.2 (16.4)	<.0001
Age group, n (%)				
20–44	422,356	21,718 (5.14)	400,638 (94.86)	
45–64	690,421	32,465 (4.70)	657,956 (95.30)	<.0001
65+	669,745	18,170 (2.71)	65,1575 (97.29)	
Sex, n (%)				<.0001
Female	940,666	43,294 (4.60)	897,372 (95.40)	
Male	841,777	29,057 (3.45)	812,720 (96.55)	
Unknown	79	2 (2.53)	77 (97.47)	
Race, n (%)				<.0001
White	1,274,293	42,596 (3.34)	1,231,696 (96.66)	
Black	133,643	9242 (6.92)	124,401 (93.08)	
Hispanic	32,244	4097 (12.71)	28,147 (87.29)	
Asian	31,568	1921 (6.09)	29,647 (93.91)	
Native American	1493	272 (18.22)	1221 (81.78)	
Multi	8271	1167 (14.11)	7104 (85.89)	
Other	17,918	750 (4.19)	17,168 (95.81)	
Unknown/Undetermined	270,669	12,130 (4.48)	258,539 (95.52)	
Not Entered	12,423	177 (1.42)	12,246 (98.58)	
Region, n (%)				<.0001
Midwest	310,124	18,037 (5.82)	292,087 (94.18)	
Northeast	490,293	21,371 (4.36)	468,922 (95.64)	
South	674,821	23,697 (3.51)	651,124 (96.49)	
West	307,284	9248 (3.01)	298,036 (96.99)	
BMI, kg/m^2				<.0001
Mean (SD)	27.5 (1.4)	27.8 (1.3)	27.5 (1.4)	
CCI Score				<.0001
Mean (SD)	0.7 (1.3)	0.7 (1.3)	0.7 (1.2)	
CCI Category, n (%)				0.0046
0	1,164,358	46,643 (4.01)	1,117,715 (95.99)	
1	325,337	14,652 (4.50)	310,685 (95.50)	
2	153,713	5205 (3.39)	148,508 (96.61)	
3	69,305	2592 (3.74)	66,713 (96.26)	
4	30,786	1194 (3.88)	29,592 (96.12)	
5+	39,023	2067 (5.30)	36,956 (94.70)	

[a]*P* values for coded vs non-coded patients; t-test for continuous variables, chi-square test for categorical variables.
BMI, body mass index; CCI, Charlson comorbidity index; SD, standard deviation.
