# Supplementary material for: Use of International Classification of Diseases, Ninth Revision Codes for Obesity: Trends in the United States from an Electronic Health Record-Derived Database
Source: Popul Health Manag. 2018 Jun 1;21(3):222–30. doi: 10.1089/pop.2017.0092 (PMC5984561; doi:10.1089/pop.2017.0092)
Supplement: Supplemental data [file Supp_Table2.pdf]

Supplementary Table S2. Prevalence of Select Comorbidities and Obesity Coding Prevalence, Patients with Body Mass Index 25–29 kg/m^2 (Overweight) and Existing Comorbidities at Index Body Mass Index[a]

Comorbidity	n	*Patients with BMI 25–29 kg/m^2 (n = 1,782,522)*		P *value*[d]
		Comorbidity[b] *prevalence, %*	*Prevalence of overweight coding*[c] *within comorbidity category, %*	
Any CVD	768,523	43.1	4.0	<.0001
—Hypertension	594,914	33.4	4.2	<.0001
—Other CVD	459,255	25.8	3.3	<.0001
Dyslipidemia	663,780	37.2	4.7	<.0001
GERD	253,867	14.2	4.6	<.0001
Malignancy	247,289	13.9	4.0	0.3607
T2DM	192,659	10.8	5.2	<.0001
Depression	187,647	10.5	5.5	<.0001
Osteoarthritis	171,638	9.6	3.4	<.0001
Vitamin D Deficiency	147,258	8.3	6.2	<.0001
Chronic Kidney Disease	74,912	4.2	4.4	<.0001
Prediabetes	68,712	3.9	7.5	<.0001
Sleep Apnea	52,449	2.9	4.6	<.0001
Gallbladder Disease	19,807	1.1	4.8	<.0001
Dyspepsia	14,578	0.8	5.3	<.0001
Inflammatory Bowel Diseases	13,137	0.7	2.9	<.0001
NAFLD	12,261	0.7	7.7	<.0001
HIV	7598	0.4	10.2	<.0001
Acute/Chronic Pancreatitis	4818	0.3	3.8	0.3215
Metabolic Syndrome	4752	0.3	8.3	<.0001
Anorexia	3002	0.2	3.6	0.1696
Cushing Syndrome	362	0.02	5.8	0.0949
Cachexia	137	0.008	10.2	0.0004
Feeding Difficulties	194	0.01	0.0	0.9561
Prader-Willi Syndrome	38	0.002	5.3	0.7069

[a]Index BMI = first recorded BMI measurement during the study period.
[b]Comorbidity confirmed by existing diagnosis ±3 month window around index BMI.
[c]ICD-9 codes for overweight: 278.02.
[d]*P* values for coded vs non-coded patients; t-test for continuous variables, chi-square test for categorical variables.
BMI, body mass index; CVD, cardiovascular disease; GERD, gastroesophageal reflux disease; HIV, human immunodeficiency virus; NAFLD, non-alcoholic fatty liver disease; T2DM, type 2 diabetes mellitus.
