# Supplementary material for: Use of International Classification of Diseases, Ninth Revision Codes for Obesity: Trends in the United States from an Electronic Health Record-Derived Database
Source: Popul Health Manag. 2018 Jun 1;21(3):222–30. doi: 10.1089/pop.2017.0092 (PMC5984561; doi:10.1089/pop.2017.0092)
Supplement: Supplemental data [file Supp_Table3.pdf]

Supplementary Table S3. Odds Ratios and 95% Confidence Intervals from Logistic Regression Model Estimating the Probability of Being Coded for Overweight, Patients with Body Mass Index 25–29 kg/m^2

	Odds ratio	*95% CI*	P *value*[a]
Demographic Characteristics			
Age (ref = 65+)			
20–44	1.909	1.860–1.959	<.0001
45–64	1.632	1.597–1.668	<.0001
Female (ref = Male)	1.289	1.267–1.312	<.0001
Race (ref = White)			
Asian	1.742	1.660–1.827	<.0001
Black	1.960	1.913–2.008	<.0001
Hispanic	4.117	3.973–4.266	<.0001
Indian (American)	5.814	5.082–6.651	<.0001
Multi	4.110	3.856–4.381	<.0001
Other	1.207	1.120–1.300	<.0001
Geographic Location (ref = West)			
Midwest	2.137	2.075–2.201	<.0001
Northeast	1.402	1.361–1.443	0.8365
South	1.281	1.246–1.317	<.0001
Clinical characteristics			
CCI Category (ref = 0)			
1	1.060	1.035–1.085	<.0001
2	0.876	0.844–0.908	<.0001
3	0.897	0.852–0.944	0.0050
4	0.938	0.870–1.011	0.6460
5+	0.947	0.876–1.022	0.8821
Comorbidities			
HIV	2.136	1.907–2.393	<.0001
Metabolic Syndrome	1.870	1.672–2.090	<.0001
Prediabetes	1.652	1.596–1.710	<.0001
Dyslipidemia	1.496	1.466–1.527	<.0001
NAFLD	1.487	1.376–1.607	<.0001
Cachexia	1.346	0.629–2.880	0.4439
Vitamin D Deficiency	1.313	1.278–1.348	<.0001
Depression	1.256	1.226–1.287	<.0001
T2DM	1.196	1.160–1.232	<.0001
Chronic Kidney Disease	1.164	1.107–1.225	<.0001
Cushing Syndrome	1.157	0.704–1.901	0.5661
Sleep Apnea	1.147	1.095–1.202	<.0001
GERD	1.059	1.035–1.084	<.0001
Dyspepsia	1.052	0.964–1.147	0.2550
Gallbladder Disease	1.048	0.972–1.129	0.2237
Hypertension	1.043	1.021–1.066	<.0001
Malignancy	1.011	0.985–1.038	0.4074
Osteoarthritis	0.852	0.826–0.879	<.0001
CVD	0.781	0.764–0.799	<.0001
Acute/Chronic Pancreatitis	0.771	0.654–0.909	0.0019
Anorexia	0.749	0.603–0.931	0.0093
Inflammatory Bowel Disease	0.735	0.658–0.821	<.0001
Prader Willi Syndrome	0.663	0.090–4.894	0.6866
Feeding Difficulties	<0.001	<0.001->999.999	0.7988

[a]From regression analysis.

CCI, Charlson comorbidity index; CI, confidence interval; CVD, cardiovascular disease; GERD, gastroesophageal reflux disease; HIV, human immunodeficiency virus; NAFLD, non-alcoholic fatty liver disease; T2DM, type 2 diabetes mellitus.
