# Supplementary material for: Use of International Classification of Diseases, Ninth Revision Codes for Obesity: Trends in the United States from an Electronic Health Record-Derived Database
Source: Popul Health Manag. 2018 Jun 1;21(3):222–30. doi: 10.1089/pop.2017.0092 (PMC5984561; doi:10.1089/pop.2017.0092)
Supplement: Supplemental data [file Supp_Fig1.pdf]

Supplementary Data

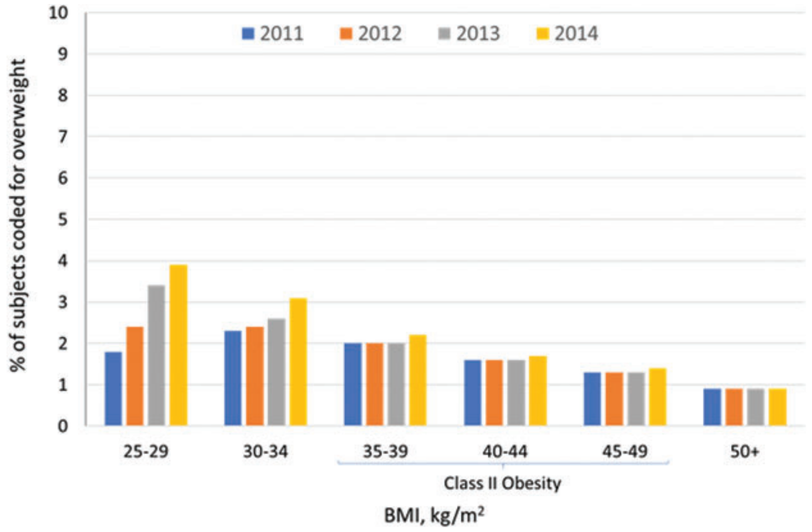

SUPPLEMENTARY FIG. S1. Prevalence of coding for overweight[a] by index body mass index[b] category, by year, 2011 to 2014.[c]

[a]ICD-9 code for overweight (278.02) captured ±3 months from date of index BMI in each year.

[b]Index BMI = first recorded BMI measurement during the study period.

[c]2014 data reflect 9 months (January – September).

BMI, body mass index; ICD-9, *International Classification of Diseases, Ninth Revision.*
