# Supplementary material for: Use of International Classification of Diseases, Ninth Revision Codes for Obesity: Trends in the United States from an Electronic Health Record-Derived Database
Source: Popul Health Manag. 2018 Jun 1;21(3):222–30. doi: 10.1089/pop.2017.0092 (PMC5984561; doi:10.1089/pop.2017.0092)
Supplement: Supplemental data [file Supp_Table4.pdf]

Supplementary Table S4. Prevalence of *International Classification of Diseases, Ninth Revision* Coding for Overweight[a] by Index Body Mass Index[b] by Year and Sex

	% of patients with ICD-9 codes for overweight[a]							
	2011		*2012*		*2013*		*2014*	
BMI, kg/m^2	*Females*	*Males*	*Females*	*Males*	*Females*	*Males*	*Females*	*Males*
25.0 – 29.9	2.1	1.5	2.7	2.0	3.8	2.9	4.4	3.4
30.0 – 34.9	2.4	2.1	2.6	2.2	2.8	2.4	3.3	2.8
35.0 – 39.9	2.0	2.0	2.0	2.0	2.0	2.0	2.3	2.2
40.0 – 44	1.6	1.6	1.6	1.6	1.6	1.7	1.7	1.7
45.0 – 49.9	1.3	1.3	1.3	1.3	1.3	1.3	1.4	1.4
≥50.0	0.9	1.0	0.9	1.0	0.9	0.9	1.0	0.9

[a]ICD-9 code for overweight: 278.02.
[b]Index BMI = first recorded BMI measurement during the study period.
BMI, body mass index; ICD-9, *International Classification of Diseases, Ninth Revision*.
